# Pronoia or reverse paranoid delusion: A brief exploration into a conspiracy in your favour

**DOI:** 10.1192/j.eurpsy.2021.2030

**Published:** 2021-08-13

**Authors:** S. Jesus, A. Costa, P. Garrido, J. Alcafache

**Affiliations:** Psiquiatria E Saúde Mental, Centro Hospitalar do Baixo Vouga, Aveiro, Portugal

**Keywords:** psychopathology, Paranoia, nosology, pronoia

## Abstract

**Introduction:**

Pronoia is a neologism originally coined in 1982 to describe a state of mind that is, in essence, the positive counterpart of paranoia. It is characterized by feeling that the world is conspiring on behalf of the person experiencing pronoia.

**Objectives:**

Brief literature review.

**Methods:**

The authors review the available literature on pronoia and present a broad overview of its description and defining characteristics. An initial search utilizing key health journal databases revealed a scarcity in available documents, therefore a generalized search utilizing the search engine Google Scholar was performed with the term “pronoia”. Relevant articles obtained from the respective bibliographic references were also consulted.

**Results:**

The primary outcome of this work is a summary of the available literature in order to build a more comprehensive understanding on pronoia. All relevant information was collated to form a cohesive description of the condition and its characteristics. We address a gap in the literature by offering a description of the lesser prevalent concept of pronoia.

**Conclusions:**

Our results demonstrate a scarcity in the available literature describing the pronoia phenomenon when compared to its well-documented counterpart, paranoia. Further exploration into this topic is merited so as to close the gap on paranoia’s lesser-known positive counterpart. By signalling the existence of this concept, we strive to contribute to an increased identification of a concept that is many times underdiagnosed due to a lack of attention to its existence.

**Disclosure:**

No significant relationships.

